# Interdisciplinary Implementation of a Synoptic Reporting Template for Melanoma Nodal Surveillance Ultrasound

**DOI:** 10.1245/s10434-024-15630-0

**Published:** 2024-07-02

**Authors:** Kelsey B. Montgomery, Zoey N. Duncan, Ashley M. Holder, Constantine M. Burgan, Samuel J. Galgano, Kristy K. Broman

**Affiliations:** 1 Department of Surgery, University of Alabama at Birmingham, Birmingham, AL USA; 2 Heersink School of Medicine, University of Alabama at Birmingham, Birmingham, AL USA; 3 Department of Surgical Oncology, University of Texas MD Anderson Cancer Center, Houston, TX USA; 4 Department of Radiology, University of Alabama at Birmingham, Birmingham, AL USA; 5 Institute for Cancer Outcomes and Survivorship, University of Alabama at Birmingham, Birmingham, AL USA; 6 Birmingham Veterans Affairs Medical Center, Birmingham, AL USA

**Keywords:** Melanoma, Sentinel lymph node positive, Nodal surveillance, Surveillance ultrasound, Synoptic reporting

## Abstract

**Background:**

With nodal surveillance increasingly used for sentinel lymph node-positive (SLN+) melanoma following the Second Multicenter Selective Lymphadenectomy Trial (MSLT-II), high-quality nodal ultrasonography (U/S) has become a critical need. Previous work has demonstrated low utilization of MSLT-II U/S criteria to define abnormal lymph nodes requiring intervention or biopsy. To address this gap, an evidence-based synoptic template was designed and implemented in this single-center study.

**Methods:**

Sentinel lymph node-positive patients undergoing nodal surveillance at a tertiary cancer center from July 2017 to June 2023 were identified retrospectively. Ultrasound reporting language was analyzed for MSLT-II criteria reported and clinically actionable recommendations (e.g., normal, abnormal with recommendation for biopsy). Following a multidisciplinary design process, the synoptic template was implemented in January 2023. Postimplementation outcomes were evaluated by using U/S reports and provider surveys.

**Results:**

A total of 337 U/S studies were performed on 94 SLN+ patients, with a median of 3 U/S per patient (range 1–12). Among 42 synoptic-eligible U/S performed postimplementation, 32 U/S (76.0%) were reported synoptically. Significant increases were seen in the number of MSLT-II criteria reported (Pre 0.5 ± 0.8 vs. Post 2.5 ± 1.0, *p* < 0.001), and clinically actionable recommendations for abnormal findings (Pre 64.0% vs. Post 93.0%, *p* = 0.04). Nearly all surgeon and radiologist survey respondents were “very” or “completely” satisfied with the clinical utility of the synoptic template (90.0%).

**Conclusions:**

Following implementation of a synoptic template, U/S reports were significantly more likely to document MSLT-II criteria and provide an actionable recommendation, increasing usefulness to providers. Efforts to disseminate this synoptic template to other centers are ongoing.

Nodal surveillance has become a mainstay in the management of sentinel lymph node-positive (SLN+) cutaneous melanoma following two multicenter randomized controlled trials: the Second Multicenter Selective Lymphadenectomy Trial (MSLT-II), and German Dermatologic Cooperative Oncology Group Trial (DeCOG-SLT).^1,2^These practice-changing trials demonstrated equivalent melanoma-specific survival for nodal surveillance compared with the prior standard of care, completion lymph node dissection (CLND), and led to the widespread adoption of nodal surveillance, rates of which have been cited at 80–96% among SLN+ melanoma patients in multiple retrospective multicenter studies.^3–5^ While nodal surveillance offers the opportunity for reduced CLND-related morbidity, it requires intensive follow up at 4- to 6-month intervals for 5 years after surgery and necessitates the availability of high-quality nodal basin ultrasound (U/S) imaging, both of which can present challenges in real-world settings.

One such real-world challenge is the application of MSLT-II nodal surveillance ultrasonography criteria, which delineate specific U/S findings associated with nodal recurrence and standardized thresholds for lymph node (LN) biopsy based on these criteria.^1^ These U/S criteria from the trial protocol were a LN length:depth ratio less than 2, hypoechoic LN center, failure to identify a nodal hilar vessel, and focal rounded area of low level echogenicity with increased vascularity.^1^ Suspicion of nodal recurrence on U/S was defined in the MSLT-II protocol as having two or more of these criteria, although participating sites were not required to externally report U/S findings.^1^ In prior work evaluating the reporting of these criteria in an early post-MSLT-II cohort of SLN+ melanoma patients undergoing nodal surveillance, MSLT-II criteria were rarely reported, and although the majority of reports included a clinically actionable recommendation, this was less consistent when U/S findings were abnormal, leaving providers uncertain regarding next steps in management.^6^ Given the rapid implementation of this evidence-based practice, concerns around the fidelity and consistency of nodal surveillance U/S in nontrial settings have been raised.^7^

Addressing gaps in the quality of nodal surveillance U/S reporting is an important step toward providing effective nodal surveillance in this population. Using quality improvement methodology, this study aimed to address this gap in nodal surveillance U/S reporting quality by evaluating the clinical utility of a synoptic reporting template for melanoma nodal surveillance U/S at a large tertiary cancer center.

## Methods

### Quality Improvement Intervention

**Fig. 1:**
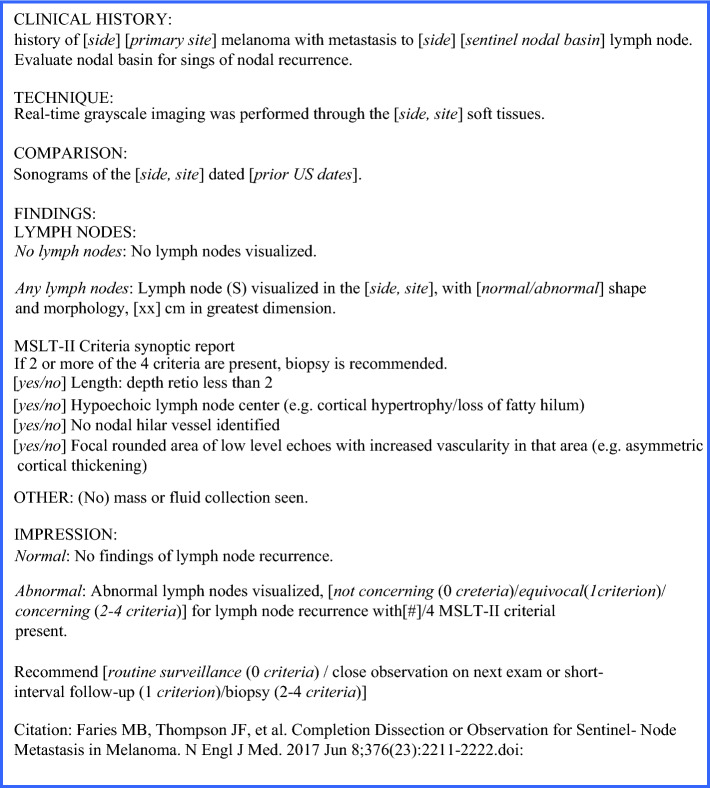
Melanoma nodal basin ultrasound synoptic reporting template

**Fig. 2:**
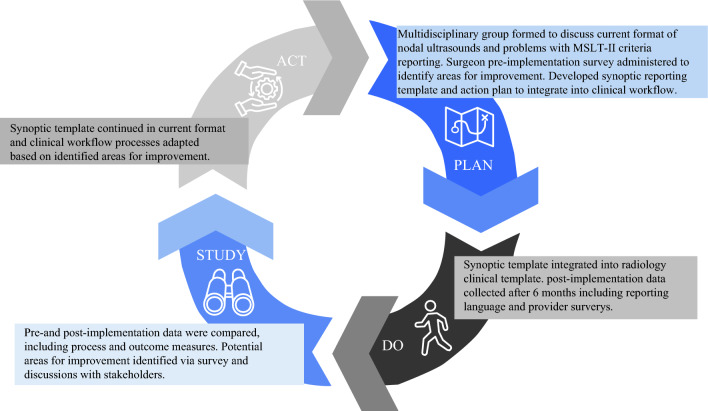
Plan-Do-Study-Act cycle. *MSLT-II* Second Multicenter Selective Lymphadenectomy Trial

Following an initial evaluation of current institutional melanoma nodal basin U/S reporting as previously described and a pre-implementation survey among melanoma providers (surgical oncologists, otolaryngologists, and surgical APPs), a group of surgical oncologists and radiologists at our tertiary cancer center was assembled to discuss the design and implementation of a synoptic reporting template for melanoma nodal ultrasonography based on MSLT-II U/S criteria for nodal recurrence.^6^ A draft synoptic reporting template was then circulated among surgeons, surgical advanced practice providers (APPs), radiologists, and sonographers to solicit initial feedback on structure and content. Additionally, brief pre-implementation meetings were held with the surgical faculty and APPs, as well as among radiology faculty and sonographers, to increase awareness of the template and share the reasoning behind its implementation. Based on stakeholder feedback, a Plan-Do-Study-Act quality improvement cycle was planned to incorporate this synoptic template (Fig. 1) into the clinical workflow and evaluate its impact on the reporting of nodal surveillance criteria (Fig. 2).

### Study Design and Cohort Selection

Approval was obtained from the University of Alabama at Birmingham Institutional Review Board. Standards for Quality Improvement Reporting Excellence (SQUIRE 2.0) guidelines were used in the reporting of this study.^8^ Following the quality improvement process described above, the synoptic reporting template was implemented into the institutional clinical workflow in January 2023. U/S report data were abstracted retrospectively for the pre-implementation period and followed prospectively for 6 months post-implementation. Abstracted variables included adherence with the synoptic reporting template (for post-implementation studies), number of MSLT-II U/S criteria reported (up to 3 criteria), whether U/S findings were normal or abnormal (1 or more MSLT-II criteria concerning for nodal recurrence), and presence of a clinically actionable recommendation (e.g., biopsy, close interval follow-up, or continue routine surveillance). Among SLN+ patients who underwent SLN biopsy between July 2017 and December 2022, all nodal surveillance U/S from July 2017 to December 2022 (pre-implementation period) and January 2023 to June 2023 (post-implementation period) were included. Nodal basin U/S studies conducted for diagnostic (i.e., not surveillance) indications were excluded.

Post-implementation surveys were conducted among surgeons/surgical APPs and radiologists to evaluate the implementation of the synoptic reporting template and to identify potential opportunities for future iterative improvement. These surveys included provider demographic characteristics and 5-point Likert scales to assess respondents’ satisfaction with the synoptic template, its clinical utility, ease of use (among radiologists) and likelihood of following the provided recommendations (among surgeons/surgical APPs).

### Study Measures

The primary outcome measure was the number of MSLT-II U/S criteria reported per nodal surveillance U/S study; this was compared pre- and post-intervention. A secondary outcome measure was the frequency of clinically actionable recommendations provided, which was compared between pre- and post-intervention periods. The primary process measure was the proportion of nodal surveillance U/S studies using the synoptic reporting template in the post-implementation period; reports were eligible for this measure if they were performed on or after January 1, 2023 and lymph nodes were visualized on U/S. Balancing measures included surgeon/surgical APP satisfaction with the clinical utility of U/S reports (via survey), and radiologist perceptions of time to complete the reports, ease of use, and clinical utility (via survey).

### Statistical Analysis

Descriptive statistics were performed for U/S report data, as well as for the surgeon and radiologist surveys. Bivariate analyses were conducted using chi-squared tests (for categorical data) and Student’s *t*-tests (for continuous data) to evaluate differences between pre- and post-intervention groups, as well as between U/S reports with normal versus abnormal findings. Monthly results of average MSLT-II criteria reported per U/S study were plotted on a U chart to evaluate variation in this outcome measure pre- and post-intervention. Statistical analyses were conducted using R version 4.3.0 (R Foundation for Statistical Computing, Vienna, Austria) and RStudio (Posit Software, Boston, MA) statistical software.^9,10^

## Results

### Descriptive Statistics

Across the full study period, 337 nodal surveillance U/S studies were performed on 94 SLN+ melanoma patients (median 3 ultrasound studies, interquartile range [IQR] 1–5). These patients had a median age of 62 years (IQR 49–69 years), were predominantly Non-Hispanic White (88 people, 93.6%), and were evenly split between male and female sex (47 patients, 50% each). Among an overall cohort of 157 SLN+ patients who underwent SLN biopsy without immediate CLND during the study period, the median follow up time was 21 months. The overall U/S adherence rate was 32.0% (defined as 1 or more U/S studies per 6-month follow up interval), although this improved significantly in the latter study years (56.5% for patients who underwent SLN biopsy in 2021–2022 vs. 18.3% for 2017–2020). An axillary nodal basin was the most common site for SLN+ (57, 60.6%), with next most common nodal basins being inguinal (18, 19.1%), cervical (14, 14.9%), multiple basins (4, 4.3%), and epitrochlear (1, 1.1%). U/S report descriptive statistics are summarized in Table 1. The majority of U/S studies had normal findings reported (264, 78.3%) compared with those reporting abnormal findings (73, 21.7%), and the majority reported clinically actionable recommendations (315 reports, 93.5%) across the entire study period.

**Table 1:** Descriptive statistics. Variables represented as frequency (percentage) for categorical variables and median (interquartile range) for continuous variables

Variable	Surveillance ultrasound reports (n = 337), n (%)
Ultrasound reports per patient (n = 94 patients), median (IQR)	3 (1, 5)
Implementation status	
Pre-implementation	291 (86.4)
Post-implementation	46 (13.6)
Ultrasound findings	
Normal	264 (78.3)
Abnormal	73 (21.7)
MSLT-II criteria reported	
0 criteria	212 (62.9)
1 criterion	54 (16.0)
2 criteria	25 (7.4)
3 criteria	46 (13.6)
Actionable recommendation	
Yes	315 (93.5)
No	22 (6.5)

*MSLT-II* Second Multicenter Selective Lymphadenectomy Trial

### Process and Outcome Measures

For the primary process measure evaluating the proportion of post-implementation U/S studies using the synoptic reporting template, 46 U/S reports (13.6%) were performed in the post-implementation period and 42 were eligible for synoptic reporting (4 studies were ineligible because of inability to visualize lymph nodes). Of these 42 eligible U/S studies, 32 reports used the synoptic template for an adherence rate of 76.2% among synoptic-eligible studies.

**Fig. 3:**
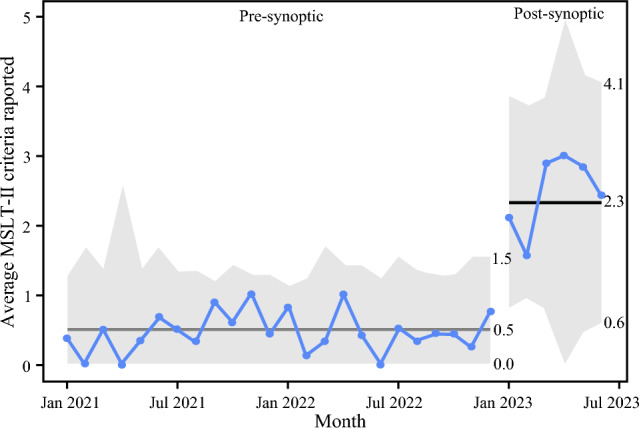
U-chart of average MSLT-II ultrasound criteria reported on a per-monthly basis. Includes 2 years pre-synoptic template and 6 months post-implementation of the synoptic template. Median values are summarized with dark grey horizontal lines through each data series, with separate values in the pre- and post-implementation periods

The number of MSLT-II criteria reported per U/S study had a statistically significant increase from a pre-implementation median of 0.0 criteria (IQR 0.0, 1.0) to a post-implementation median of 3.0 criteria (IQR 2.3-3.0) reported out of a 3-criteria maximum (*p* < 0.001) (Table 2). A quality improvement U chart demonstrating this increase in the average number of MSLT-II criteria reported per U/S on a monthly basis is depicted in Fig. 3. There also was an increase in the proportion of reports with clinically actionable recommendations, from 92.8% (270/291 pre-implementation reports) to 97.8% (45/46 post-implementation reports), although this was not statistically significant (*p* = 0.3). There was no statistically significant difference in the number of U/S with abnormal findings (*p* = 0.12), but the proportion of abnormal findings did increase from 20.3 to 30.4% (59/291 vs. 14/46).

**Table 2:** Comparison of pre- versus post-implementation reporting of MSLT-II criteria and actionable recommendations

Variables	Pre-implementation reports (n = 291), n (%)	Post-implementation reports (n = 46), n (%)	*p*
Synoptic report used			
Yes		32 (69.6)	N/A
No		10 (21.7)	
N/A	291 (100)	4 (8.7)	
Ultrasound criteria reported			
Lymph node length:depth ratio	45 (15.5)	36 (78.3)	**< 0.001**
Hypoechoic lymph node center	46 (15.8)	32 (69.6)	
Lymph node hilar vascularity	47 (16.2)	36 (78.3)	
Total criteria reported			
0 criteria	192 (68.6)	5 (11.9)	**< 0.001**
1 criterion	53 (18.9)	1 (2.4)	
2 criteria	20 (7.1)	5 (11.9)	
3 criteria	15 (5.4)	31 (73.8)	
Ultrasound findings			
Normal	232 (79.7)	28 (69.6)	0.1
Abnormal	59 (20.3)	14 (30.4)	
Actionable recommendation (all reports, n = 333)			
Yes	270/291 (92.8)	45/46 (97.8)	0.3
No	21/291 (7.2)	1/46 (2.2)	
Actionable recommendation (abnormal findings, n = 73)			
Yes	38/59 (64.0)	13/14 (92.9)	**0.04**
No	21/59 (36.0)	1/14 (7.1)	

Statistically significant values are given in bold

### Balancing Measures

Before integration of the synoptic template into the clinical workflow, a pre-implementation survey was distributed to melanoma surgeons and surgical APPs. Five participants completed the survey. These participants included both surgical oncology- and otolaryngology-trained surgeons, most of whom performed five or more SLN biopsies for melanoma per month. All participants either agreed (4/5, 80.0%) or strongly agreed (1/5, 20.0%) that surveillance U/S findings influenced the likelihood that they obtained a LN biopsy. However, the majority of participants stated that they only sometimes (4/5, 80.0%) understood the appropriate next steps for a patient based on the U/S report, and most were only moderately satisfied (2/5, 40.0%) or somewhat satisfied (2/5, 40.0%) with the clinical utility of the original U/S reporting style.

Six months after the implementation of the synoptic reporting template, post-implementation surveys were completed by four surgeons or surgical APPs (surgery post-implementation survey), and six radiology faculty or residents (radiology post-implementation survey). Among the surgery providers, all participants strongly agreed (3/4, 75.0%) or agreed (1/4, 25.0%) that the synoptic format allowed them to identify findings that were relevant, and they always (2/4, 50.0%) or often (2/4, 50.0%) understood the appropriate next steps for the patient based on the U/S report. They were completely (1/4, 25.0%) or very satisfied (3/4, 75.0%) with the clinical utility of the synoptic reports and found the updated format to be a significant (3/4, 75.0%) or moderate improvement (1/4, 25.0%) compared with prior. Radiology respondents were asked to compare the synoptic template to the prior format and found the synoptic template to be somewhat (5/6, 83.3%) or very effective (1/6, 16.7%) for ease of use. The majority found it to be very effective (5/6, 83.3%) for clinical utility. There were no concerns with the time to complete the synoptic reports, and four respondents (66.7%) were more confident in making clinical recommendations. Overall, the majority of radiology respondents were very (4/6, 66.7%) or completely satisfied (1/6, 16.7%) with the synoptic reporting template.

## Discussion

While the shift away from routine CLND to nodal surveillance for SLN+ melanoma has been rapid compared with the often tepid pace of evidence-based practice implementation, development of institutional processes to support and optimize nodal surveillance in real-world settings is ongoing. Our prior work demonstrated that nodal basin U/S, which are required at frequent intervals for 5 years after surgery, can vary widely in their quality and specificity for evaluating MSLT-II criteria for nodal recurrence.^6,7^ Following implementation of a collaboratively-designed synoptic template for melanoma nodal U/S at our large tertiary cancer center, significant improvements were seen in both the number of MSLT-II criteria reported and surgical provider and radiologist satisfaction with the clinical utility of nodal U/S reports.

While equivalent melanoma-specific survival between nodal surveillance and CLND was achieved in the closely monitored trial setting of MSLT-II, barriers to the delivery of consistent, high-quality nodal surveillance present challenges to achieving similar results in real-world settings. One such barrier includes loss to follow-up or difficulty achieving consistent adherence to ultrasound surveillance, which has been previously reported as ranging from 34% in a single-center study up to 83% for the highest-adherence center in a multi-institutional study (study range 35–83%, overall 58% U/S adherence).^3,11^ These rates are likely reflective of an early implementation period for these new surveillance practices, as evidenced by the improved adherence rates seen in the cohort presented in this study. Specific to nodal surveillance U/S, concerns over the quality and interpretability of nodal U/S reports have been previously identified. In a qualitative study from Mott et al. in which surgical and medical oncologists were interviewed, multiple participants reported the perception that radiologists may not be aware of MSLT-II criteria or the reasoning behind melanoma nodal surveillance and thus may (unknowingly) omit the melanoma-specific clinically relevant findings.^7^ Given the frequency with which SLN+ patients are undergoing nodal surveillance in lieu of CLND, as high as 80–96% of SLN+ patients in multiple post-MSLT-II multicenter studies,^3–5^ interventions to address this issue could benefit a significant proportion of patients with SLN+ melanoma.

After considering these concerns about reliability and consistency with the reporting of MSLT-II criteria, a synoptic reporting template seemed an ideal choice for improving melanoma nodal U/S reports when this need was identified at our institution.^6^ Synoptic reporting tools have become increasingly common across the spectrum of cancer care, including radiology synoptic reporting tools, such as the Breast Imaging Reporting and Data System (BI-RADS) and Thyroid Imaging Reporting and Data System (TI-RADS), as well as synoptic operative reports and pathology reports.^12–15^ Across these different disciplines, the primary benefits of the synoptic format have included improved communication between users (e.g., pathologist or radiologist and surgeon or medical oncologist), clearer recommendations for next steps, and improved documentation of relevant information for future clinical or research use.^16–19^ Results from this study demonstrate similar outcomes with a nodal U/S synoptic reporting template, which enabled surgeon/surgical APP survey respondents to identify relevant findings and understand appropriate next steps in management. Furthermore, radiologist respondents expressed greater confidence in making clinical recommendations using the clearly delineated MSLT-2 criteria without imposing additional burdens on their time. As the population of SLN+ melanoma patients undergoing nodal surveillance continues to expand post-MSLT-II, having succinctly communicated findings from the radiology to surgical teams should enable the consistent delivery of high-quality surveillance care and represents a primary benefit of this synoptic template.

Facilitating factors to the implementation of this synoptic reporting template included having surgeon and radiologist “champions” who were recognized leaders in their division/sections and who participated in the design of the synoptic template, solicited feedback, and encouraged use of the synoptic template. This collaborative team also created a consistent line of communication between departments that aided in identifying issues and developing solutions in real time. In terms of developing the synoptic template, aligning the clinical recommendations to a point-based system with the same criteria used in the MSLT-II trial (e.g., if 2 or more U/S criteria were seen, biopsy is recommended) helped ground the radiologists’ recommendations in the evidence source that the surgeons were most familiar with and already using to guide their nodal surveillance practices.

Several logistical barriers were identified during the initial implementation, some of which have already been addressed in subsequent PDSA cycles. As many of the patients receiving nodal U/S in the post-implementation period had undergone previous nodal U/S as part of their nodal surveillance, occasionally the prior non-synoptic reports would be “copy-forwarded” and updated with current findings but without the synoptic reporting language. This was addressed through education with the radiology sonographers and a custom addition of the synoptic template to the “menu” of interpretation templates available for soft tissue U/S studies, so that the synoptic template could be auto-imported into the text of the report draft. This mechanism also helped to address issues with use of the synoptic report across different anatomical nodal basins; sometimes a neck U/S performed for melanoma nodal surveillance would focus on evaluation of the thyroid, for example, and less on the cervical nodal basin of interest. During the implementation period, stakeholders also identified a need to locate and scan the sentinel lymph node biopsy scar. This was especially pertinent for head and neck melanomas because of their variable drainage patterns to areas, such as occipital and post-auricular basins, which may not routinely be included in a cervical nodal U/S that focuses on anterior (level I-V) nodes. Further iterative improvements to the use of the synoptic report are ongoing, as well as efforts to collaborate with other institutions to share the synoptic reporting template.

Limitations of this study that may affect its interpretation and generalizability include its single-institution retrospective design, as well as barriers and facilitators to implementation of the synoptic reporting template that may be institution-specific. Of note, while U/S criteria for nodal recurrence were specified by the MSLT-II study protocol for participating sites, there was no requirement for synoptic or other external reporting of U/S criteria during MSLT-II; therefore, comparison of findings from this U/S protocol with patterns of nodal recurrence has not been specifically studied in a randomized, controlled fashion. Additionally, while findings represent the early post-implementation period, this intervention will need to be evaluated over a longer period of time in future work to ensure its continued success and identify ongoing opportunities for improvement. While this strategy was feasible at our institution based on the current radiology workflow and the support of departmental project champions, we recognize that adaptations may be needed for other institutions to implement a similar intervention.

## Conclusions

Following a collaborative quality improvement process with a multidisciplinary team, a synoptic reporting template for melanoma nodal surveillance ultrasound in SLN+ melanoma was successfully implemented at our tertiary cancer center. This synoptic template improved the consistency of nodal U/S reporting and the subjective utility of the report among users. Efforts are ongoing to distribute the synoptic template to other institutions and enact iterative process improvements based on stakeholder feedback.
